# An *N*-Linked Bidentate Phosphoramidite Ligand (*N*-Me-BIPAM) for Rhodium-Catalyzed Asymmetric 1,4-Addition of Arylboronic Acids to α,β-Unsaturated Ketones

**DOI:** 10.3390/molecules18010014

**Published:** 2012-12-20

**Authors:** Yasunori Yamamoto, Kazunori Kurihara, Yoshinori Takahashi, Norio Miyaura

**Affiliations:** Frontier Chemistry Center, Faculty of Engineering, Hokkaido University, Sapporo 060-8628, Japan

**Keywords:** asymmetric conjugate addition, rhodium catalyst, bidentate phosphoramidite ligand

## Abstract

A new bidentate phosphoramidite (*N*-Me-BIPAM) based on Shibasaki’s *N*-linked BINOL was synthesized. This ligand appears to be highly effective for rhodium-catalyzed asymmetric conjugated addition of arylboronic acids to α,β-unsaturated enones. The reaction of *ortho*-substituted arylboronic acid with acyclic and cyclic enones provides the corresponding products in good yields and enantioselectivities.

## 1. Introduction

Metal-catalyzed conjugated addition reactions of carbon nucleophiles to α,β-unsaturated compounds are the most widely used reactions for asymmetric carbon-carbon bond formation [1,2,3]. Much interest has recently been shown in rhodium-catalyzed conjugate addition of arylboronic acids to α,β-unsaturated carbonyl compounds [1,2,3,4,5,6,7,8] using various ligands [9,10,11,12,13,14,15,16,17,18,19,20,21] such as biaryl bisphosphines [4,5,6,7,8], phosphoramidites [22,23,24,25,26,27,28], diphosphonite [29], amidomonophosphines [30,31], *N*-heterocyclic carbenes [32,33], P-chiral phosphine [34], and dienes [35,36,37,38,39,40,41,42,43]. Although many chiral ligands give adducts with good selectivity for cyclic enones, there are few ligands that give good results for both acyclic and cyclic enones. In addition, though conjugate addition of *ortho*-substituted arylboronic acids to α,β-unsaturated cyclic enones has been achieved in high enantioselectivities [9,10,11,12,13,14,15,16,17,18,19,20,21,44,45,46,47,48,49,50,21,44], there have been few reports on this reaction for acyclic enones [38,51,52]. On the other hand, we have already reported that a new bidentate phosphoramidite ligand (Me-BIPAM) based on *O*-linked-BINOL was synthesized and that a rhodium/Me-BIPAM complex was a better catalyst than several monodentate phosphoramidites for conjugate addition of arylboronic acids to α,β-unsaturated cyclic and acyclic carbonyl compounds [53,54]. However, we were not satisfied with the enantioselectivities for acyclic enones such as (*E*)-3-nonene-2-one using Me-BIPAM. Therefore, we reported that the bidentate phosphoramidite *N*-Me-BIPAM, which was newly synthesized on the basis of *N*-linked-BINOL [55], was highly efficient for rhodium-catalyzed asymmetric arylation of *N*-sulfonyl aldimine with arylboronic acids [56].

Herein we report that rhodium/*N*-Me-BIPAM catalyzed 1,4-addition of arylboronic acids to α,β-unsaturated enones. *N*-Me-BIPAM was found to be highly effective for rhodium-catalyzed conjugate addition of arylboronic acids to α,β-unsaturated acyclic and cyclic enones. Furthermore, the use of *N*-Me-BIPAM was necessary to achieve high enantioselectivity for the reaction of *ortho*-substituted arylboronic acids to acyclic and cyclic enones.

## 2. Results and Discussion

**Scheme 1:**
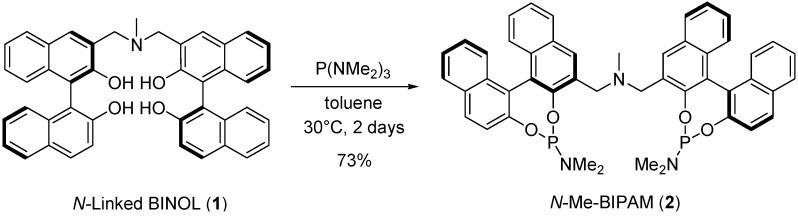
Synthesis of bisphosphoramidite (*N*-Me-BIPAM).

*N*-linked bidentate phosphoramidite was easily synthesized from Shibasaki’s *N*-linked BINOL and P(NMe_2_)_3_ in good yield (Scheme 1). ^31^P-NMR of the mixture of Rh(acac)(C_2_H_4_)_2_ and *N*-Me-BIPAM exhibited a single signal at 160.0 ppm (d, *J*_Rh-P_ = 292.3 Hz), suggesting the intramolecular complexation of two phosphorous atoms to a rhodium metal center. The formation of a 1:1 complex was also confirmed by mass spectroscopy (ESI), which showed a molecular weight of 976.2152 (M+H).

The efficiency of *N*-Me-BIPAM was investigated in rhodium-catalyzed conjugate addition of arylboronic acids to enones. We first examined the addition of phenylboronic acid to (*E*)-3-nonene-2-one in an aqueous solvent (Table 1). Preparation of the rhodium catalyst from [Rh(coe)_2_Cl]_2_, *N*-Me-BIPAM and a base in 1,4-dioxane or in DME resulted in low selectivity (entries 1–5). Among the representative inorganic bases employed, K_2_CO_3_ was found to be the best, giving 87% *ee* (entry 4). However, the use of triethylamine for a combination of [Rh(nbd)_2_]BF_4_ and *N*-Me-BIPAM resulted in a quantitative yield with 92% *ee* (entry 7).

**Table 1:** Optimization of the reaction conditions. 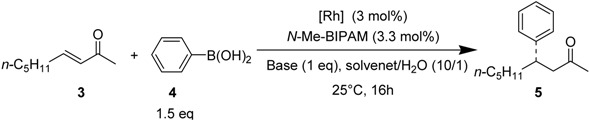

Entry	[Rh]	Solvent	Base	Yield (%) ^a^	*ee* (%) ^b^
1	[Rh(coe)_2_Cl]_2_	dioxane	KOH	96	78
2	[Rh(coe)_2_Cl]_2_	DME	KOH	99	81
3	[Rh(coe)_2_Cl]_2_	DME	K_3_PO_4_	59	73
4	[Rh(coe)_2_Cl]_2_	DME	K_2_CO_3_	94	87
5	[Rh(coe)_2_Cl]_2_	DME	NEt_3_	trace	-
6	[Rh(nbd)_2_]BF_4_	DME	K_2_CO_3_	57	87
7	[Rh(nbd)_2_]BF_4_	DME	NEt_3_	99	92

^a^ Isolated yield; ^b^ Determined by HPLC.

With these optimized conditions, 1,4-addition of arylboronic acids to representative α,β-unsaturated acyclic and cyclic enones was carried out in the presence of a [Rh(nbd)_2_]BF_4_/*N*-Me-BIPAM catalyst (Table 2). *N*-Me-BIPAM achieved high enantioselectivities in the range of 92–95% *ee* for (*E*)-3-nonen-2-one (entries 1–3). The selectivities were in the range of 82–90% *ee* for (*E*)-5-methyl-3-hexen-2-one (entries 4–6). However, this ligand was not effective for substrates having a phenyl group at the carbonyl carbon or the β-carbon due to steric hindrance of the aryl ring (entries 7 and 8).

**Table 2:** Asymmetric conjugated addition of arylboronic acids to α,β-unsaturated enones. 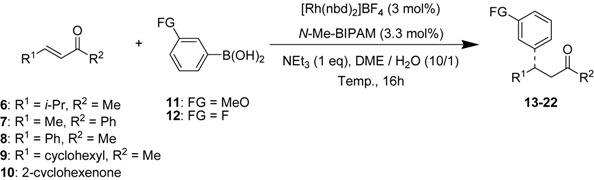

Entry	Enones	FG	Temp. (°C)	Product	Yield (%) ^a^	*ee* (%) ^b^
1	(*E*)-C_5_H_11_CH=CHCOCH_3_	H	25	13	99	92 (*S*)
2 ^c^	(*E*)-C_5_H_11_CH=CHCOCH_3_	MeO	25	14	96	94 (*S*)
3	(*E*)-C_5_H_11_CH=CHCOCH_3_	F	50	15	83	95 (+)
4	(*E*)-(CH_3_)_2_CHCH=CHCOCH_3_	H	50	16	83	87 (*R*)
5	(*E*)-(CH_3_)_2_CHCH=CHCOCH_3_	MeO	25	17	60	90 (*R*)
6	(*E*)-(CH_3_)_2_CHCH=CHCOCH_3_	F	50	18	42	82 (+)
7	(*E*)-CH_3_CH=CHCOPh	MeO	50	19	87	77 (+)
8	(*E*)-PhCH=CHCOCH_3_	MeO	50	20	99	77 (+)
9 ^c^	(*E*)-cyclo-C_6_H_11_CH=CHCOCH_3_	MeO	50	21	94	88 (+)
10 ^c^	2-Cyclohexenone	H	50	22	99	99 (*R*)

^a^ Isolated yield; ^b^ Determined by HPLC; ^c^ Used [Rh(coe)_2_Cl]_2_, KOH and dioxane/H_2_O instead of optimized condition.

**Scheme 2:**
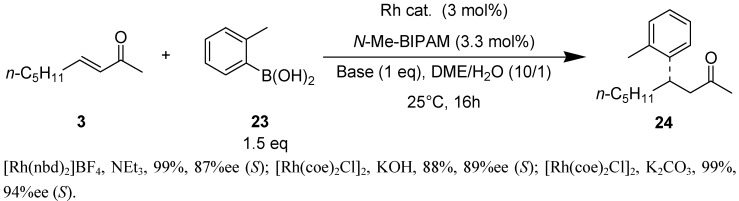
Asymmetric 1,4-addition of 2-tolylboronic acids to 3-nonene-2-one.

The steric hindrance of an *ortho*-substituent on the arylboronic acids slows down the reaction rate significantly and decreases the enantioselectivity. It was interesting that *N*-Me-BIPAM exhibited good performance for such boronic acids. The results of 1,4-addition of 2-tolylboronic acid to (*E*)-3-nonen-2-one are shown in Scheme 2. The use of neutral [Rh(coe)_2_Cl]_2_ and K_2_CO_3_ for *N*-Me-BIPAM resulted in a quantitative yield and the best selectivity (94% *ee*).

Table 3 shows the results of 1,4-addition of arylboronic acids possessing a methyl, fluoro or methoxy group at the *ortho*-carbon or 1-naphthylboronic acid to the representative acyclic and cyclic enones. The use of a series of arylboronic acids for (*E*)-3-nonen-2-one showed enantioselectivities decreasing in the order of F > Me > 1-naphthyl > MeO (entries 1–5). On the other hand, the effect of substituents was F > MeO > 1-naphthyl > Me for (*E*)-CH_3_CH=CHCOPh (entries 6–9). Thus, the selectivities were greatly dependent on the bulkiness and electronic property of substituted arylboronic acids and enone substrates. Among them, 1,4-addition of *ortho*-tolylboronic acid to (*E*)-3-nonen-2-one resulted in 92% yield and 97% *ee* with 0.1 mol% catalyst loading (entry 2). When benzylideneacetone which have the bulky substitution on the β-position was used as a substrate, the product was obtained in good enantioselectivity, but reactivity was lower (entry 10). There was no difficulty in achieving high enantioselectivities for 5- and 6- and 7-membered enones at room temperature (entries 11–16). Most of the reactions resulted in more than 94% *ee* for these cyclic substrates.

**Scheme 3:**
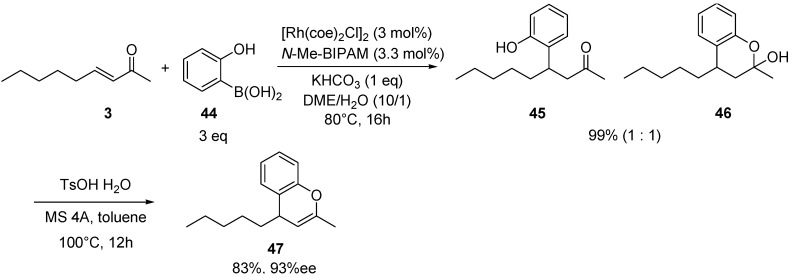
Synthesis of 2-methyl-4-pentyl-4*H*-chromene.

The good performance of *N*-Me-BIPAM for ortho-substituted arylboronic acids was applied to the synthesis of optically active 4-alkyl-4H-chromenes (Scheme 3). Under conditions optimized in Scheme 3 using K_2_CO_3_ as the base, 1,4-addition of 2-hydroxyphenylboronic acid resulted in very low yield due to hydrolytic B-C bond cleavage with water. However, the use of 3 equivalents of boronic acid and KHCO_3_ at 80 °C afforded an 1,4-adduct in quantitative yield. The 1,4-adduct thus obtained was a 1:1 mixture of ketone and hemiacetal. It was then treated with TsOH·H_2_O and MS 4A in toluene at 100 °C [57] to give optically active 2-methyl-4-pentyl-4H-chromene in 78% yield with 98% *ee* [58].

**Table 3:** Asymmetric conjugated addition of *o*-substituted arylboronic acids to α,β-unsaturated enones. 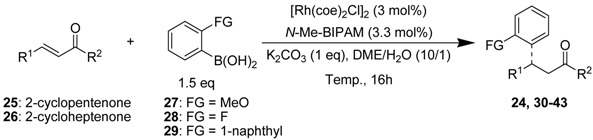

Entry	Enones	FG	Temp. (°C)	Product	Yield (%) ^a^	*ee* (%) ^b^
1	(*E*)-*n*-C_5_H_11_CH=CHCOCH_3_	Me	25	24	99	94 (*S*)
2	(*E*)-*n*-C_5_H_11_CH=CHCOCH_3_	Me	50	24	92	97 ^c^ (*S*)
3	(*E*)-*n*-C_5_H_11_CH=CHCOCH_3_	MeO	50	30	86	80 (+)
4	(*E*)-*n*-C_5_H_11_CH=CHCOCH_3_	F	50	31	90	99 (+)
5	(*E)-n*-C_5_H_11_CH=CHCOCH_3_	1-naphtyl	80	32	77	86 (+)
6	(*E*)-CH_3_CH=CHCOPh	Me	50	33	80	81 (−)
7	(*E*)-CH_3_CH=CHCOPh	MeO	50	34	88	92 (+)
8	(*E*)-CH_3_CH=CHCOPh	F	50	35	86	99 (−)
9	(*E*)-CH_3_CH=CHCOPh	1-naphtyl	50	36	87	83 (−)
10	(*E*)-PhCH=CHCOCH_3_	Me	50	37	49	90 (*R*)
11	2-Cyclopentenone	Me	25	38	90	97 (*R*)
12	2-Cyclopentenone	MeO	25	39	67	94 (*R*)
13	2-Cyclopentenone	F	25	40	32	97 (*R*)
14	2-Cyclopentenone	1-naphtyl	25	41	77	99 (*R*)
15	2-Cyclopentenone	Me	25	42	94	87 (*R*)
16	2-Cycloheptenone	Me	25	43	92	97 (*R*)

^a^ Isolated yield; ^b^ Determined by HPLC; ^c^ Used 0.1 mol% of rhodium catalyst.

**Figure 1:**
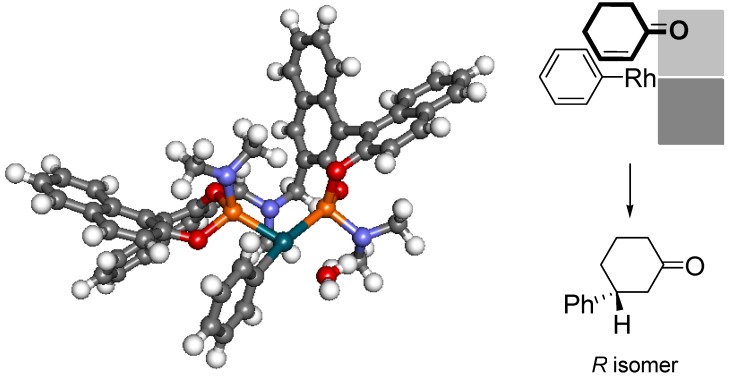
Optimized structure of [Rh(Ph)(H_2_O)((*R,R*)-*N*-Me-BIPAM)].

Finally, a stable conformation of the [Rh(Ph)(H_2_O)((*R,R*)-*N*-Me-BIPAM)] intermediate generated by transmetalation of phenylboronic acid to [Rh(OH)(H_2_O)((*R,R*)-*N*-Me-BIPAM)] was calculated on the basis of a theoretical method (B3LYP/6-31G++(d)/B3LYP/LANL2DZ level). There is a completely planar coordination space in the upper and lower left areas consisting of a phenyl group on a rhodium atom. A naphthoxy group in the upper right area occupies a pseudo-axial position and an NMe_2_ group in the lower right area occupies a pseudo-equatorial position, thus suggesting that the space is accessible to reactants in the upper right quadrant and two quadrants in the left area. On the basis of this calculation, a mode of coordination of an enone to the phenyl rhodium(I) intermediate is proposed in Figure 1. The *re*-coordination of a substrate can be preferred without significant steric interaction for giving the experimentally observed *R* enantiomer by parallel coordination of the C-O double bond to the Ph-Rh bond for the next insertion process. On the other hand, the coordination of an enone from its opposite *si* face is blocked by the equatorial NMe_2_ group.

## 3. Experimental

### 3.1. General

^1^H-NMR spectra were recorded on a JEOL ECX-400 (400 MHz) in CDCl_3_ with tetramethylsilane as an internal standard. Chemical shifts are reported in part per million (ppm), and signal are expressed as singlet (s), doublet (d), triplet (t), quartet (q), multiplet (m), and broad (br). ^13^C-NMR spectra were recorded on a JEOL ECX-400 (100 MHz) in CDCl_3_ (δ_C_ = 77.0) with tetramethylsilane as an internal standard. Chemical shifts are reported in part per million (ppm). HPLC analysis was directly performed with chiral stationary phase column, Chiralpak AD-H, IB or Chiralcel OD-H, OB-H purchased from DAICEL Co., Ltd. (Osaka, Japan). High resolution mass spectra (HRMS) were recorded on a JEOL JMS 700TZ mass spectrometer at the Center for Instrumental Analysis, Hokkaido University, Japan. Optical rotations were measured on a HORIBA SEPA-300 digital polarimeter. Kanto Chemical silica gel 60N (particle size 0.063-0.210 mm) was used for flash column chromatography.

### 3.2. Synthesis of N-Me-BIPAM (**2**)

(*R,R*)-3,3'-[Methyliminobis(methylene)]bis(1,1'-binaphtylene-2,2'-diol) (**1**, 2.4 mmol) and P(NMe_2_)_3_ (6 mmol) in dry toluene were stirred for 2 days at 30 °C under nitrogen. The crude solid obtained by evaporation of the solvent was purified by column chromatography to give *N*-Me-BIPAM (**2**) as a white solid (73%). [α]^22^_D_ = −578.3 (c 0.56, CHCl_3_),^1^H-NMR (400 MHz, CD_2_Cl_2_) δ = 8.32 (s, 2H), 7.93 (q, *J* = 9.1 Hz, 6H), 7.36–7.41 (m, 4H), 7.18–7.29 (m, 8H), 4.09 (d, *J* = 15.4 Hz, 2H), 3.76 (d, *J* = 15.4 Hz, 2H), 2.58 (s, 3H), 2.32 (s, 6H), 2.30 (s, 6H), ^13^C-NMR (CD_2_Cl_2_) δ = 149.5, 149.4, 148.6, 132.9, 131.5, 130.9, 129.8, 129.3, 128.2, 127.6, 127.3, 126.9, 126.4, 125.5, 124.8, 124.0, 122.7, 122.6, 121.1, 57.3, 44.5, 36.1, 34.8 ^31^P-NMR (100 MHz, CD_2_Cl_2_) 149.5 HRMS (FAB) calcd for C_47_H_42_N_3_O_4_P_2_ (M+H) 774.2651, Found 774.2667.

### 3.3. General Procedure for Asymmetric 1,4-Addition

A flask charged with rhodium catalyst (0.015 mmol) and *N*-Me-BIPAM (0.033 mmol) was flushed with nitrogen. Solvent (3 mL) was then added. After being stirred for 1 h at room temperature, aryl boronic acid (1.5 mmol), enone (1 mmol), base (1 mmol) and water (0.3 mL) were added. The resulting mixture was stirred for 16 h at 25 °C, 50 °C or 80 °C. The mixture was extracted with ethyl acetate, washed with brine, and dried over MgSO_4_. After concentration, the residue was purified by column chromatography on silica gel with hexane/ethyl acetate to give the product as a clear liquid.

The spectral data of compounds **13** [59], **14** [59], **15** [54], **16** [59], **17** [59], **18** [54], **20** [54], **21** [54], **22** [59], **24** [38], **38** [29], **39** [44], **40** [25], **41** [45], **42** [47], **43** [32] was previously reported. The specific rotations of these compounds were (*S*)-**13** ([α]^23^_D_ = +15.2 (c 0.11, CHCl_3_)), (*S*)-**14** [([α]^23^_D_ = +4.83 (c 0.30, CHCl_3_)], **15** ([α]^23^_D_ = +12.6 (c 0.70, CHCl_3_)), (*R*)-**16** [([α]^23^_D_ = +27.4 (c 0.55, CHCl_3_)], (*R*)-**17** ([α]^23^_D_ = +24.7 (c 0.33, CHCl_3_)), **18** ([α]^23^_D_ = +20.1 (c 0.53, CHCl_3_)), **20** ([α]^23^_D_ = +1.04 (c 0.60, CHCl_3_)), **21** ([α]^23^_D_ = +26.8 (c 0.41, CHCl_3_)), (*R*)-**22** ([α]^23^_D_ = +17.9 (c 0.78, CHCl_3_)), (*S*)-**24** ([α]^22^_D_ = +12.2 (c 0.51, CHCl_3_)), (*R*)-**38** ([α]^23^_D_ = +39.4 (c 0.48, CHCl_3_)), (*R*)-**39** ([α]^23^_D_ = +13.8 (c 0.38, CHCl_3_)), (*R*)-**40** ([α]^23^_D_ = +11.5 (c 0.31, CHCl_3_)), (*R*)-**41** ([α]^23^_D_ = +8.70 (c 0.25, CHCl_3_)), (*R*)-**42** ([α]^23^_D_ = +19.4 (c 0.26, CHCl_3_)), (*R*)-**43** ([α]^23^_D_ = +20.6 (c 0.30, CHCl_3_)).

*3-(3-Methoxyphenyl)-1-phenylbutan-1-one* (**19**). [α]^24^_D_ = +0.41 (c 0.48, CHCl_3_), 77% *ee* [HPLC conditions: Chiralcel OD-H column, hexane/ethanol = 9:1, flow = 0.5 mL/min, wavelength = 254 nm, tR = 29.4 min and 32.8 min]; ^1^H-NMR (CDCl_3_) δ = 1.33 (d, *J* = 7.25 Hz, 3H), 3.18 (dd, *J* = 8.2, 16.8 Hz, 1H), 3.31 (dd, *J* = 5.4, 16.8 Hz, 1H), 3.45–3.54 (m, 1H), 3.79 (s, 3H), 6.74–6.76 (m, 1H), 6.84 (t, *J* = 1.81 Hz, 1H), 6.88 (d, *J* = 7.7 Hz, 1H), 7.23 (t, *J* = 7.7 Hz, 1H), 7.42–7.46 (m, 2H), 7.53–7.56 (m, 1H), 7.94 (dd, *J* = 1.36, 7.25 Hz, 2H); ^13^C-NMR (CDCl_3_) δ = 21.9, 35.7, 47.0, 55.3, 112.1, 112.2, 119.3, 127.4, 127.9, 129.0, 129.5, 130.4, 133.1, 137.2, 148.4, 159.8, 199.1; exact mass calcd for C_17_H_18_O_2_: 254.1307; Found 254.1290.

*4-(2-Methoxyphenyl)nonan-2-one* (**30**). [α]^24^_D_ = +3.13 (c 0.40, CHCl_3_), 80% *ee* [HPLC conditions: Chiralcel OJ-H column, hexane/2-propanol = 100:1, flow = 0.3 mL/min, wavelength = 254 nm, tR = 20.5 min and 23.0 min]; ^1^H-NMR (CDCl_3_) δ = 0.80–0.84 (m, 3H), 1.10–1.25 (m, 6H), 1.53–1.64 (m, 2H), 2.04 (s, 3H), 2.64–2.75 (m, 2H), 3.52–3.59 (m, 1H), 3.81 (s, 3H), 6.83–6.89 (m, 2H), 7.10–7.16 (m, 2H); ^13^C-NMR (CDCl3) δ = 13.9, 22.4, 27.0, 30.0, 31.7, 34.6, 34.8, 49.8, 55.2, 110.5, 120.5, 127.0, 127.7, 132.4, 157.1, 208.5; exact mass calcd for C_15_H_24_O_2_: 248.1776; Found 248.1786.

*4-(2-Fluorophenyl)nonan-2-one* (**31**). [α]^22^_D_ = −3.32 (c 0.56, CHCl_3_), 99% *ee* [HPLC conditions: Chiralpak IA column, hexane/2-propanol = 200:1, flow = 0.8 mL/min, wavelength = 254 nm, tR = 21.7 min and 24.7 min]; ^1^H-NMR (CDCl_3_) δ =0.82 (d, J = 6.8 Hz, 3H), 1.08–1.32 (m, 6H), 1.57–1.62 (m, 2H), 2.05 (s, 3H), 2.76 (d, *J* = 7.3 Hz, 2H), 3.37–3.44 (m, 1H), 6.95–7.17 (m, 4H); ^13^C-NMR (CDCl_3_) δ =14.1, 22.6, 27.2, 30.4, 31.7, 35.2, 49.5, 115.6, 124.2, 127.0, 128.6, 130.0, 131.2, 207.8, 161.5; exact mass calcd for C_15_H_21_FO: 236.1576; Found 236.1576.

*4-Naphthalen-1-yl-nonan-2-one* (**32**). [α]^22^_D_ = +18.6 (c 0.54, CHCl_3_), 81% *ee* [HPLC conditions: Chiralpak IB column, hexane/2-propanol = 100:1, flow = 0.5 mL/min, wavelength = 254 nm, tR = 17.7 min and 18.9 min]; ^1^H-NMR (CDCl_3_) δ =0.80 (t, *J* = 6.7 Hz, 3H), 1.10–1.30 (m, 6H), 1.70–1.83 (m, 2H), 2.04 (s, 3H), 2.84 (d, *J* = 6.7 Hz, 2H), 4.02–4.17 (m, 1H), 7.35 (d, *J* = 7.0 Hz, 1H), 7.41–7.53 (m, 3H), 7.71 (d, *J* = 7.9 Hz, 1H), 7.85 (d, *J* = 7.9 Hz, 1H), 8.19 (d, *J* = 8.2 Hz, 1H); ^13^C-NMR (CDCl_3_) δ = 14.1, 22.6, 27.2, 30.7, 32.1, 36.2, 50.9, 123.3, 125.6, 126.1, 126.9, 129.1, 132.0, 134.2, 141.1, 208.1; exact mass calcd for C_19_H_24_O: 268.1827; Found 268.1836.

*1-Phenyl-3-o-tolylbutan-1-one* (**33**). [α]^24^_D_ = −20.1 (c 0.42, CHCl_3_), 81% *ee* [HPLC conditions: Chiralpak IB column, hexane/2-propanol = 99.8:0.2, flow = 0.5 mL/min, wavelength = 254 nm, tR = 22.4 min and 24.2 min]; ^1^H-NMR (CDCl_3_) δ = 1.31 (d, *J* = 6.8 Hz, 3H), 2.41 (s, 3H), 3.21 (dd, *J* = 8.2, 16.7 Hz, 1H), 3.30 (dd, *J* = 5.4, 16.7 Hz, 1H), 3.74–3.82 (m, 1H), 7.10–7.29 (m, 3H), 7.28 (d, *J* = 6.8 Hz, 1H), 7.46 (t, *J* = 7.7 Hz, 2H), 7.53–7.58 (m, 1H), 7.96 (d, *J* = 7.2 Hz, 2H); ^13^C-NMR (CDCl_3_) δ = 19.7, 21.5, 30.5, 46.4, 125.3, 125.4, 127.1, 127.9, 128.2, 129.5, 130.6, 132.3, 133.9, 135.4, 137.3, 144.9, 199.3; exact mass calcd for C_17_H_18_O: 238.1358; Found 238.1358.

*3-(2-Methoxyphenyl)-1-phenylbutan-1-one* (**34**). [α]^22^_D_ = +6.73 (c 0.54, CHCl_3_), 92% *ee* [HPLC conditions: Chiralpak IB column, hexane/2-propanol = 99.8:0.2, flow = 0.5 mL/min, wavelength = 254 nm, tR = 30.4 min and 58.1 min]; ^1^H-NMR (CDCl_3_) δ = 1.32 (d, *J* = 6.8 Hz, 3H), 3.03–3.09 (m, 1H), 3.37 (dd, *J* = 4.5, 15.9 Hz, 1H), 3.80–3.90 (m, 1H), 3.82 (s, 3H), 6.86 (d, *J* = 8.2 Hz, 1H), 6.94 (t, *J* = 7.3 Hz, 1H), 7.26–7.18 (m, 2H), 7.45 (t, *J* = 7.7 Hz, 2H), 7.55 (t, *J* = 7.3 Hz, 1H), 7.99 (d, *J* = 7.7 Hz, 2H); ^13^C-NMR (CDCl_3_) δ = 19.9, 29.7, 46.1, 55.4, 110.6, 120.7, 127.3, 127.5, 127.8, 128.1, 129.1, 129.4, 132.2, 133.8, 134.5, 137.3, 156.9, 199.8; exact mass calcd for C_17_H_18_O_2_: 254.1307 ; Found 254.1317.

*3-(2-Fluorophenyl)-1-phenylbutan-1-one* (**35**). [α]^23^_D_ = −2.91 (c 0.53, CHCl_3_), 95% *ee* [HPLC conditions: Chiralpak IB column, hexane/2-propanol = 99.8:2, flow = 0.5 mL/min, wavelength = 254 nm, tR = 22.2 min and 24.2 min]; ^1^H-NMR (CDCl_3_) δ = 1.35 (d, *J* = 6.8 Hz, 3H), 3.21 (dd, *J* = 8.2, 16.8 Hz, 1H), 3.38 (dd, *J* = 5.9, 16.8 Hz, 1H), 3.71–3.80 (m, 1H), 6.98–7.09 (m, 2H), 7.14–7.20 (m, 1H), 7.27 (ddt, *J* = 1.36, 1.81, 7.7 Hz, 1H), 7.44 (t, *J* = 7.7 Hz, 2H), 7.54 (dd, *J* = 7.3, 7.7 Hz, 1H), 7.95 (dd, *J* = 1.4, 7.3 Hz, 2H); ^13^C-NMR (CDCl_3_) δ = 20.7, 29.9, 45.4, 115.7, 123.5, 125.5, 127.0, 127.4, 127.9, 129.0, 129.5, 132.3, 134.0, 137.1, 160.9, 198.9; exact mass calcd for C_16_H_15_FO: 242.1107; Found 242.1119.

*3-Naphthalen-1-yl-1-phenylbutan-1-one* (**36**). [α]^22^_D_ = −56.3 (c 0.51, CHCl_3_), 83% *ee* [HPLC conditions: Chiralpak IB column, hexane/2-propanol = 99.8:0.2, flow = 0.5 mL/min, wavelength = 254 nm, tR = 47.3 min and 63.0 min]; ^1^H-NMR (CDCl_3_) δ = 1.50 (d, *J* = 6.8 Hz, 3H), 3.32–3.45 (m, 2H), 4.39–4.48 (m, 1H), 7.44–7.59 (m, 7H), 7.75 (dd, *J* = 4.5, 5.0 Hz, 1H), 7.89 (d, *J* = 8.2 Hz, 1H), 7.98 (d, *J* = 7.7 Hz, 2H), 8.22 (d, *J* = 8.6 Hz, 1H); ^13^C-NMR (CDCl_3_) δ = 21.2, 29.7, 46.8, 122.6, 123.3, 125.2, 125.7, 126.2, 127.1, 127.4, 128.2, 128.7, 129.0, 129.9, 131.2, 132.4, 134.0, 137.3, 142.7, 199.5; exact mass calcd for C_20_H_18_O: 274.1358; Found 274.1358.

*(R)-4-Phenyl-4-o-tolylbutan-2-one* (**37**). [α]^24^_D_ = −66.6 (c 0.52, CHCl_3_), 90% *ee* [HPLC conditions: Chiralcel OD-H column, hexane/ethanol = 9:1, flow = 0.5 mL/min, wavelength = 254 nm, tR = 14.8 min and 16.8 min]; ^1^H-NMR (CDCl_3_) δ = 2.07 (s, 3H), 2.30 (s, 3H), 3.15 (d, *J* = 7.2 Hz, 2H), 4.78 (t, *J* = 7.2 Hz, 1H), 7.09–7.13 (m, 2H), 7.13–7.20 (m, 4H), 7.20–7.24 (m, 3H); ^13^C-NMR (CDCl_3_) δ = 19.8, 30.7, 41.9, 50.0, 126.0, 126.3, 126.3, 126.4, 127.9, 128.5, 130.8, 136.4, 141.5, 143.5, 206.9; exact mass calcd for C_17_H_18_O: 238.1358; Found 238.1373.

*2-Methyl-4-pentyl-4H-chromene* (**47**). [α]^22^_D_ = +282.1 (c 0.70, THF), 98% *ee* [HPLC conditions: Chiralcel OD-H column, hexane/ethanol = 100:1, flow = 0.5 mL/min, wavelength = 254 nm, tR = 7.5 min and 8.6 min]; ^1^H-NMR (CD_2_Cl_2_) δ = 0.84 (t, *J* = 6.8 Hz, 3H), 1.17–1.33 (m, 6H), 1.51–1.59 (m, 2H), 1.87 (s, 3H), 3.38 (d, *J* = 5.4 Hz, 1H), 4.70 (d, *J* = 4.1 Hz, 1H), 6.81–6.83 (m, 1H), 6.94–6.99 (m, 1H), 7.07–7.11 (m, 1H); ^13^C-NMR (CD_2_Cl_2_) δ = 13.9, 19.1, 23.9, 31.4, 33.9, 39.5, 99.9, 115.8, 122.8, 124.6, 126.9, 128.4, 147.5, 151.9; exact mass calcd for C_15_H_20_O: 216.1514; found 216.1517.

## 4. Conclusions

We have synthesized a new bidentate phosphoramidite, *N*-Me-BIPAM, based on Shibasaki’s *N*-linked BINOL. This ligand was found to be an excellent ligand for both cyclic and acyclic enones. Due to its low electron-donating property, the reactions were completed in a shorter time at room temperature than that of traditional BINAP complexes. Furthermore, *N*-Me-BIPAM allowed the 1,4-addition of *ortho*-substituted arylboronic acid to acyclic and cyclic enones with high enantioselectivities.
